# Antioxidants and the risk of stroke: results from NHANES and two-sample Mendelian randomization study

**DOI:** 10.1186/s40001-024-01646-5

**Published:** 2024-01-12

**Authors:** Rundong Chen, Hanchen Liu, Guanghao Zhang, Qian Zhang, Weilong Hua, Lei Zhang, Nan Lv, Yilei Zhang, Dongwei Dai, Rui Zhao, Qiang Li, Qinghai Huang, Yi Xu, Pengfei Yang, Jianmin Liu, Qiao Zuo

**Affiliations:** 1 Neurovascular Center, Changhai Hospital, Naval Medical University, #168 Changhai Road, Shanghai, 200433 China; 2 School of Health Science and Engineering, University of Shanghai for Science and Technology, Shanghai, China; 3 Department of Neurosurgery, Sir Run Run Shaw Hospital, College of Medicine, Zhejiang University, Zhejiang, China; 4 Nursing Department, Sir Run Run Shaw Hospital, Zhejiang University School of Medicine, Zhejiang, China

**Keywords:** Antioxidants, Retinol, Selenium, Stroke, Subarachnoid hemorrhage, NHANES, Mendelian randomization

## Abstract

**Background:**

Stroke is the second leading cause of death worldwide, and observational studies have suggested a correlation between antioxidants and reduced stroke risk. However, it remains unclear whether causal relationships exist.

**Methods:**

This study first performed a cross-sectional study of the association between the Composite Dietary Antioxidant Index (CDAI) and stroke using data from the National Health and Nutrition Examination Survey (NHANES) 2007–2018. Second, a two-sample univariable Mendelian Randomization (MR) was performed to analyze the causal effect of circulating levels of antioxidants on different subtypes of stroke.

**Results:**

The cross-sectional study included a total of 24,892 participants representing more than 200 million US non-institutionalized residents, a multivariable logistic regression model revealed that the risk of stroke decreased by 3.4% for each unit increase in CDAI (*P* = 0.017), with a non-linear association found, indicating a reduction in stroke risk before an inflection point of 3.078. MR analysis revealed that genetically determined levels of retinol had a suggestive protective effect on subarachnoid hemorrhage (SAH) (OR = 0.348, *P* = 0.025), and genetically determined levels of selenium had a suggestive protective effect against SAH (OR = 0.826, *P* = 0.007). However, no causal relationship was found between antioxidants and ischemic stroke or intracranial hemorrhage risk.

**Conclusions:**

Evidence suggests that diet-derived antioxidants may reduce the risk of stroke, as indicated by the protective effects of retinol and selenium against SAH. However, more research is needed to fully understand how antioxidants prevent stroke.

**Supplementary Information:**

The online version contains supplementary material available at 10.1186/s40001-024-01646-5.

## Introduction

Stroke is the second leading cause of mortality globally, and its prevalence is projected to rise due to demographic shifts. Studies have revealed that diet-derived antioxidants—often found in fruits and vegetables—have been linked with a decreased chance of experiencing stroke or cerebrovascular disease [1–8]. Joshipura et al. found that individuals with a high intake of fruits and vegetables had a relative risk of 0.69 compared to those with a low intake [7]. Additionally, the American Stroke Association recommends consuming a diet rich in fruits, vegetables, nuts, and low-fat dairy products and reducing saturated fat, as a means of primary prevention to reduce the incidence of stroke [8].

In vitro* and *in vivo animal studies have shown that various antioxidants are involved in reducing the pathophysiology process of oxidative stress in the development of stroke. Vitamin C may improve endothelial function by preventing leukocyte aggregation and adhesion to the endothelium [9]; Vitamin E can prevent oxidative stress, inflammation and endothelial dysfunction, as well as reducing circulating levels of C reactive protein [10]. The special structure of the brain makes it highly susceptible to oxidative stress during stroke [11]. Therefore, antioxidants may be an effective way to reduce the level of oxidative stress in the body and prevent stroke.

The purpose of this study is to determine whether antioxidants have a protective effect on stroke risk. We first analyzed the data from the National Health and Nutrition Examination (NHANES) to explore the possible link between the Composite Dietary Antioxidant Index (CDAI) and stroke. The CDAI is a reliable tool for measuring the overall antioxidant content of an individual’s diet [12]. We further assessed the causality between antioxidants and stroke risk using Mendelian Randomization (MR) approach [13], which, analogous to randomized controlled trials, could avoid residual confounding and was therefore able to assess the causal exposure-outcome relationship [14].

## Materials and methods

### Study design

We first performed a cross-sectional study of the association between CDAI and stroke using data from NHANES. In NHANES, a representative sample of the US noninstitutionalized population was sampled over a 2 year cycle to describe health conditions and disease burden. The data for these cross-sectional surveys were obtained from the National Center for Health Statistics (NCHS), which is accessible at https://www.cdc.gov/nchs/index.htm. The dataset was last accessed on November 1, 2023. We used the data from six consecutive 2 year cycles spanning 2007–2018. Second, the two-sample MR was performed to analyze the causal relationships of diet-derived antioxidants and different subtypes of stroke. This report was drafted in accordance with the guidelines set out by the Strengthening the Reporting of Observational Studies in Epidemiology (STROBE) for cross-sectional and MR studies. This study utilized publicly available data and was classified as exempt by the Ethics Committee of Shanghai Changhai Hospital.

### Cross-sectional study by NHANES

A total of 26,433 participants were included after excluding those who did not meet the inclusion criteria (Additional file 2: Fig S1). The primary outcome was the occurrence of stroke, which was defined as the participant who answered “yes” to the medical conditions questionnaire “Has a doctor or other health professional ever told you that you had a stroke?”. The primary exposure variable was the CDAI, which was calculated by adding the six normalized vitamins and minerals, including vitamins A, C, and E, selenium, zinc and carotenoids from food only. The diet-derived intake information was obtained from a detailed dietary interview component that estimated the types and amounts of foods and beverages consumed during the 24 h period prior to the interview. CDAI was computed by aggregating the standardized dietary antioxidant intakes, which were normalized by subtracting the sex-specific mean and dividing by the sex-specific standard deviation as follows:

$${\text{CDAI}}= \sum_{{\text{i}}=1}^{6} \frac{\mathrm{Xi }-\mathrm{ \mu i }}{{\text{Si}}}$$

We also collected covariates in order to reduce bias. Demographic variables included age, gender, race, education level, marital status, and annual household income. We tabulated the following stroke-related risk factors: body mass index (BMI), smoking status, alcohol consumption, hypertension, diabetes mellitus, hyperlipidemia, coronary heart disease and physical activity. The diagnostic criteria of these covariates were listed in Additional file 2: Table S1.

### MR analysis

The study design and three main assumptions of MR were shown in Additional file 2: Fig S2. The summary genome-wide association study (GWAS) of circulating antioxidants and stroke from published studies were analyzed, and their sources were detailed in Additional file 2: Table S2. The genetic instruments of circulating antioxidants included by CDAI were used, including vitamin A (retinol), vitamin C (ascorbate), vitamin E (α-tocopherol and γ-tocopherol), selenium and zinc. As carotenoid in human circulating was mainly present in the form of carotene, we were using GWAS data of circulating carotene as a replacement [15]. The leading single nucleotide polymorphisms (SNPs) associated with each antioxidant as instrumental variables (IVs, *P* < 5 × 10^−6^) were identified in GWAS data after linkage disequilibrium (LD distance > 10,000 kb, *r*^2^ < 0.001) was eliminated [16]. We harmonized the SNP alleles across studies and removed palindromic SNPs with ambiguous allele frequencies (0.42–0.58).

Stroke, as an outcome variable, refered to a range of conditions including ischemic stroke (IS) [17], intracranial hemorrhagic (ICH) [18] and subarachnoid hemorrhage (SAH) (Additional file 1) [19]. To further understand these conditions, different subgroups of stroke were analyzed. There were no overlapping samples between exposure cohorts and outcome cohorts to avoid bias.

### Statistical analysis

Given the complex, multistage, probability sampling design of NHANES, weights were considered in statistical analyses in this study in order to account for the unequal probabilities of selection. Taking into account that all participants enrolled had completed the day one dietary recall, the “dietary day one sample weight” was determined using the “least common denominator” rule, which was calculated as 1/6 * wtdrd1. The continuous variables were expressed as the weighted mean (SE), and the categorical parameters were expressed as N (weighted%). Student’s *t*-test was used to assess for differences between stroke and non-stroke participants, while a chi-square test was used to assess for differences in categorical variables. A multivariable logistic regression analysis was used to examine the association between CDAI and stroke. Odds ratio (OR) and 95% confidence interval (CI) were calculated to assess the strength of the association. Then, to explore the possible shape of the curve between CDAI and stroke, we further performed the analysis using the restricted cubic spine model (RCS). We selected four knots at the 5, 35, 65 and 95th quartiles. Furthermore, we conducted subgroup analyses to test the robustness and potential variations in different subgroups, and their interactions were tested. *P* < 0.05 was considered statistically significant.

The primary MR analysis was conducted by using random-effects inverse-variance weighted (IVW) regression analysis [20]. This was then followed by weighted median (WM) and MR-Egger regression analysis to verify the robustness of IVW estimate [21, 22]. In a subsequent sensitivity analysis, Cochran's IVW Q statistic was used to detect heterogeneity, intercept test of MR-Egger regression to detect horizontal pleiotropy [21], and MR pleiotropy residual sum and outlier (MR-PRESSO) to detect outliers [23]. Results were expressed as ORs with 95% CI on stroke risk per unit antioxidant change. The *F*-statistic can help assess the strength of IVs. In addition, the “leave-one-out” analysis was performed to identify potentially heterogeneous IVs by sequentially removing each instrumental IV. The significance threshold was set at 0.008 (0.05/6) according to Bonferroni correction, and *P* < 0.008 suggested a strong significance, while 0.008 < *P* < 0.05 provided suggestive evidence [24]. The statistical analysis was performed using R software (version 4.1.3).

## Results

### Cross-sectional study by NHANES

A total of 26,433 NHANES participants represented more than 200 million US non-institutional residents. Weighted baseline characteristics of participants with and without stroke were compared and listed in Table 1. Individuals with diabetes mellitus, hypertension, hyperlipidemia, and coronary artery disease were more susceptible to stroke, as were older, females, isolated, impoverished, overweight, Blacks, smokers, alcohol consumers, and those with a lower level of physical activity. The CDAI scores of individuals with stroke were lower compared to those without this affliction, taking into consideration the intake of each diet-derived antioxidant, vitamin E, zinc and selenium still have a protective effect against stroke.

**Table 1:** Weighted baseline characteristics of participants

Variable	Total	Non-stroke	Stroke	*P*-value
Age categories				< 0.0001
< 70	22046 (83.40)	21445 (88.46)	601 (58.21)	
≥ 70	4387 (16.60)	3920 (11.54)	467 (41.79)	
Gender				0.090
Female	13222 (50.02)	12694 (50.72)	528 (54.49)	
Male	13211 (49.98)	12671 (49.28)	540 (45.51)	
Race/Ethnicity				< 0.0001
Mexican American	3883 (14.69)	3791 (8.37)	92 (4.65)	
Non-Hispanic Black	5588 (21.14)	5296 (10.65)	292 (15.20)	
Non-Hispanic White	11563 (43.74)	11025 (68.18)	538 (69.70)	
Other	5399 (20.43)	5253 (12.81)	146 (10.46)	
Educational level				< 0.0001
Colleges or above	14230 (53.83)	13817 (62.80)	413 (42.11)	
Junior middle schools or below	6080 (23.00)	5734 (14.28)	346 (24.36)	
Senior high schools or GED	6123 (23.16)	5814 (22.93)	309 (33.53)	
Marital status				< 0.0001
Married/Partnered	15665 (59.26)	15105 (62.32)	560 (57.76)	
Separated/Widowed	5938 (22.46)	5520 (18.30)	418 (34.80)	
Single	4830 (18.27)	4740 (19.39)	90 (7.45)	
Annual household income				< 0.0001
< 20,000	5558 (21.03)	5174 (13.98)	384 (29.33)	
≥ 20,000	20875 (78.97)	20191 (86.02)	684 (70.67)	
Smoke status				< 0.0001
No	14392 (54.45)	13991 (55.12)	401 (39.30)	
Yes	12041 (45.55)	11374 (44.88)	667 (60.70)	
Alcohol consumption				< 0.0001
No	8056 (30.48)	7639 (25.09)	417 (36.42)	
Yes	18377 (69.52)	17726 (74.91)	651 (63.58)	
BMI				< 0.0001
< 25	7391 (27.96)	7135 (29.27)	256 (23.91)	
25–29	8670 (32.80)	8332 (32.95)	338 (28.97)	
≥ 30	10372 (39.24)	9898 (37.78)	474 (47.12)	
Hypertension				< 0.0001
No	14878 (56.29)	14680 (62.55)	198 (23.75)	
Yes	11555 (43.71)	10685 (37.45)	870 (76.25)	
Diabetes mellitus				< 0.0001
No	22601 (85.5)	21912 (89.94)	689 (68.29)	
Yes	3832 (14.50)	3453 (10.06)	379 (31.71)	
Hyperlipidemia				< 0.0001
No	7936 (30.02)	7776 (31.26)	160 (14.64)	
Yes	18497 (69.98)	17589 (68.74)	908 (85.36)	
Coronary heart disease				< 0.0001
No	25316 (95.77)	24434 (96.98)	882 (81.78)	
Yes	1117 (4.23)	931 (3.02)	186 (18.22)	
Physical activity				< 0.0001
High	16002 (60.54)	15583 (66.19)	419 (43.30)	
Low	3538 (13.39)	3402 (13.04)	136 (11.47)	
Inactive	6893 (26.08)	6380 (20.77)	513 (45.23)	
CDAI	0.28 (0.05)	0.31 (0.05)	− 0.66 (0.17)	< 0.0001
Vitamin A, μg	643.13 (9.03)	644.62 (9.21)	596.08 (27.09)	0.087
Vitamin C, mg	80.97 (1.21)	81.13 (1.18)	76.03 (5.05)	0.292
Vitamin E, mg	8.87 (0.09)	8.92 (0.08)	7.19 (0.23)	< 0.0001
Zinc, mg	11.55 (0.08)	11.61 (0.08)	9.78 (0.25)	< 0.0001
Selenium, μg	114.56 (0.63)	115.17 (0.62)	95.37 (2.61)	< 0.0001
Carotenoid, μg	9754.35 (150.53)	9779.33 (149.23)	8965.47 (589.39)	0.156

Values indicate the weighted mean (SE) or N (weighted%). *P*-values are also weighted

*CDAI* Composite Dietary Antioxidant Index

**Fig. 1:**
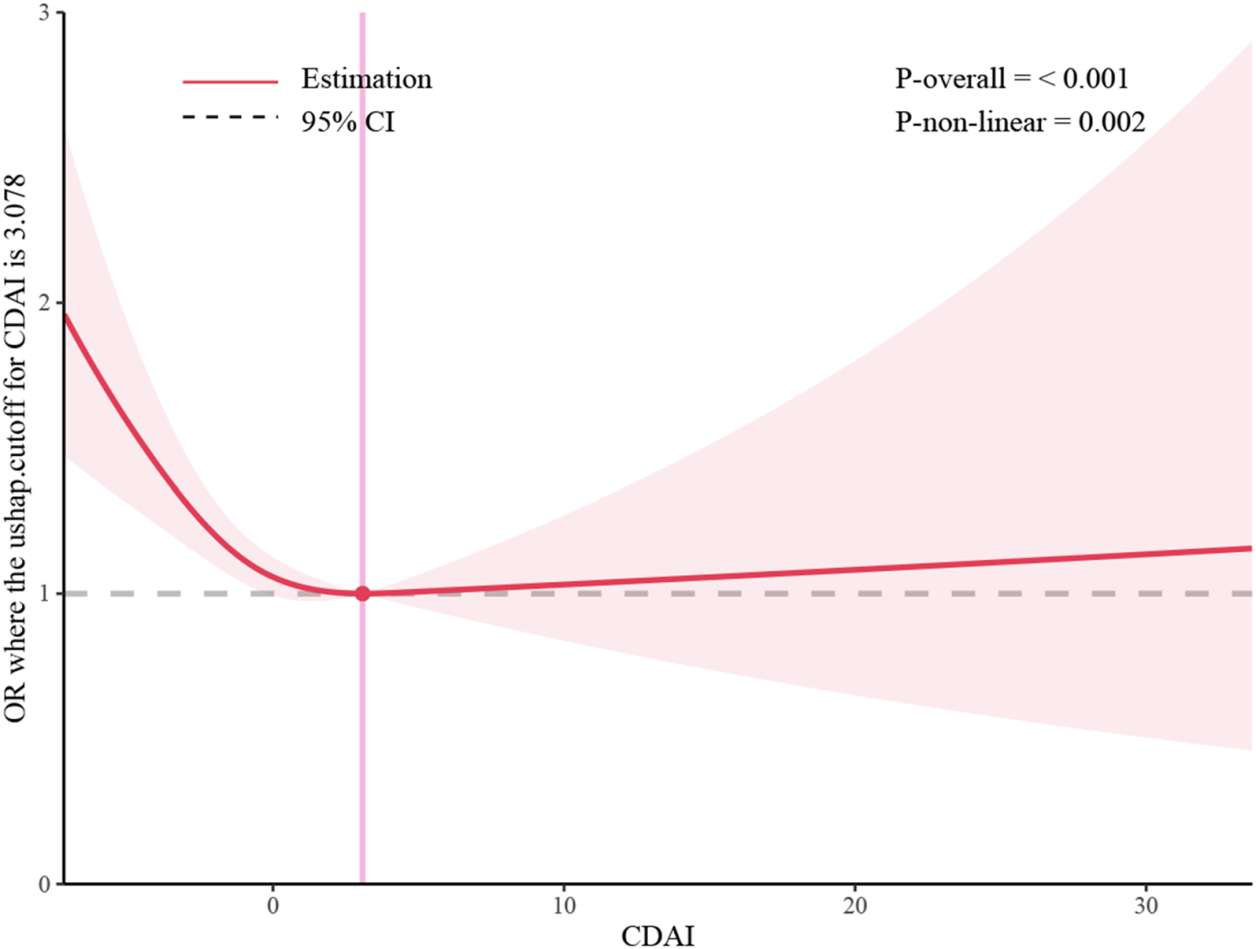
Restricted cubic spline plot of the association between CDAI and stroke. All variables are taken into account and adjusted accordingly. The shaded part represented the 95% CI. *CDAI* Composite Dietary Antioxidant Index, *OR* Odds Ratio

**Fig. 2:**
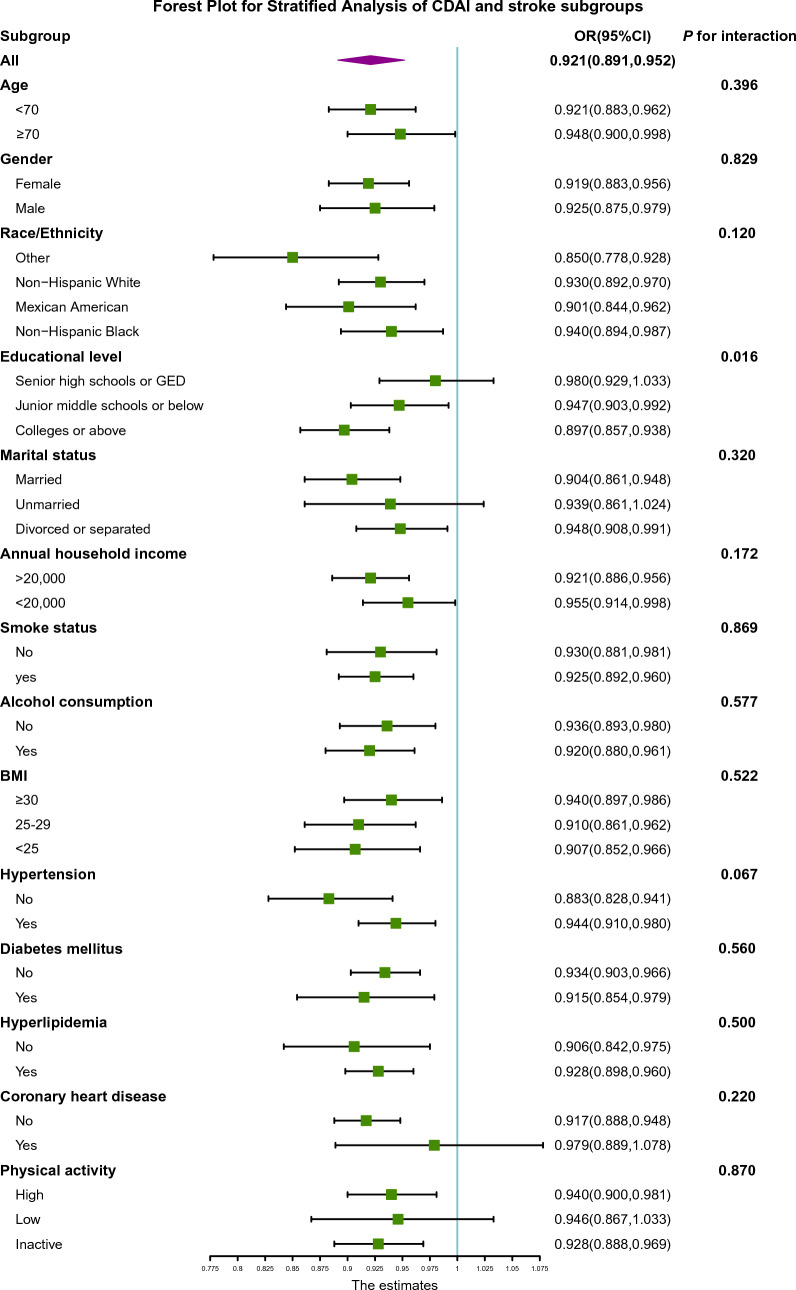
Forest plots of the association between CDAI and stroke subgroups. *CDAI* Composite Dietary Antioxidant Index, *OR* Odds ratio, *CI* Confidence interval

Three weighted models were constructed to examine the relationship between CDAI and stroke in this study (Table 2). The results of the crude model showed that for each unit increase in CDAI, the risk of stroke decreased by 8.9% (OR = 0.921, 95% CI 0.891–0.952, *P* = 0.016), and the risk of stroke decreased by 6.6% (OR = 0.934, 95% CI 0.904–0.966,* P* < 0.001) when only adjusting for demographic variables. In the fully adjusted model, the risk of stroke dropped by 3.4% for each unit increase in CDAI (OR = 0.966, 95% CI 0.937–0.997,* P* = 0.017). In the sensitivity analysis, we categorized the CDAI by quartiles for trend testing (Additional file 2: Table S3). The results of the study showed that as the CDAI level increased, the stroke risk of patients significantly decreased (*P* for trend < 0.001) (Table 2). Subsequently, in-depth investigation was conducted to explore the relationship between the six dietary antioxidants in CDAI and stroke, which revealed that vitamin E, zinc, and selenium could potentially act as the antioxidative components in CDAI, providing a protective effect against stroke after adjusting for confounding factors (Additional file 2: Table S4–S9). Then, we further conducted a restricted cubic spline (RCS) model after adjusting for other variations and found evidence of a non-linear correlation with a saturation effect between CDAI and stroke (*P* for non-linearity = 0.001) (Fig. 1). The inflection point was 3.078, and each unit increase in CDAI before the inflection point was associated with a 6.0% reduction in stroke risk (OR = 0.940, 95% CI 0.904–0.976,* P* = 0.002). However, the association is reversed after the inflection point, although it is not statistically significant (OR = 1.024, 95% CI 0.977–1.074, *P* = 0.312). Finally, we analyzed the CDAI across risk factor subgroups linked to stroke (Fig. 2). It indicated a potential interaction with educational attainment, the link between CDAI and stroke seemed most pronounced in individuals of the highest educational status. However, no significant interactions were detected for other risk factors.

**Table 2:** Association of CDAI and stroke

	Crude model^a^		Model1^b^		Model 2^c^	
	OR (95% CI)	*P*	OR (95% CI)	*P*	OR (95% CI)	*P*
CDAI	0.921(0.891, 0.952)	< 0.0001	0.934(0.904, 0.966)	< 0.0001	0.966(0.937, 0.997)	0.017
Lowest quartiles	ref					
Q2	0.684(0.544, 0.860)	0.001	0.700(0.560, 0.875)	0.002	0.753(0.596, 0.953)	0.019
Q3	0.595(0.497, 0.711)	< 0.0001	0.639(0.528, 0.773)	< 0.0001	0.793(0.651, 0.966)	0.022
Q4	0.417(0.322, 0.539)	< 0.0001	0.468(0.363, 0.603)	< 0.0001	0.612(0.472, 0.793)	< 0.001
*P* for trend^d^		< 0.0001		< 0.0001		< 0.001

*CDAI* Composite Dietary Antioxidant Index, *OR* Odds Ratio, *ref* reference

^a^Crude model: no covariates were adjusted

^b^Model 1: age, gender, and race were adjusted

^c^Model 2: age, gender, race, education level, marital status, annual household income, smoking status, alcohol consumption, BMI, diabetes mellitus, hypertension, hyperlipidemia, coronary artery disease and physical activity were adjusted

^d^Test for trend based on variable containing median value for each quintile

### MR analysis

**Fig. 3:**
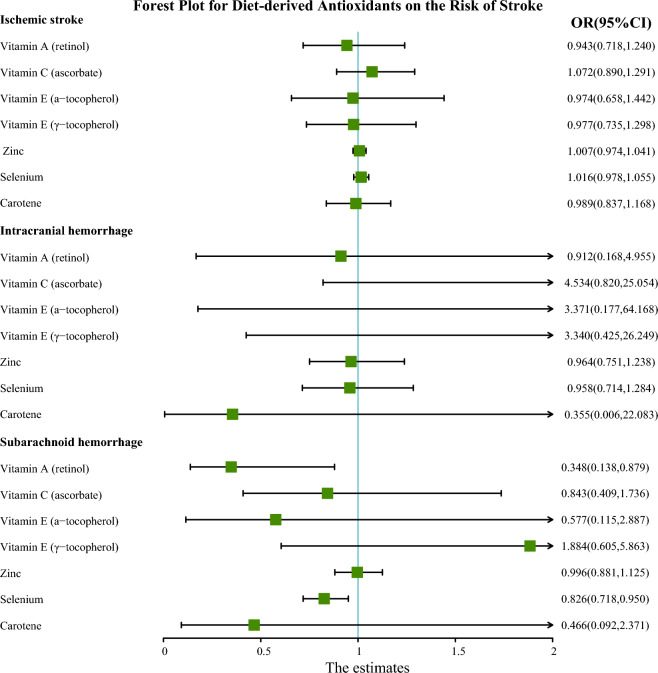
Forest plot for the causal effect of diet-derived antioxidant on the risk of stroke derived from inverse variance weighted (IVW). *OR* Odds ratio, *CI* Confidence interval

**Fig. 4:**
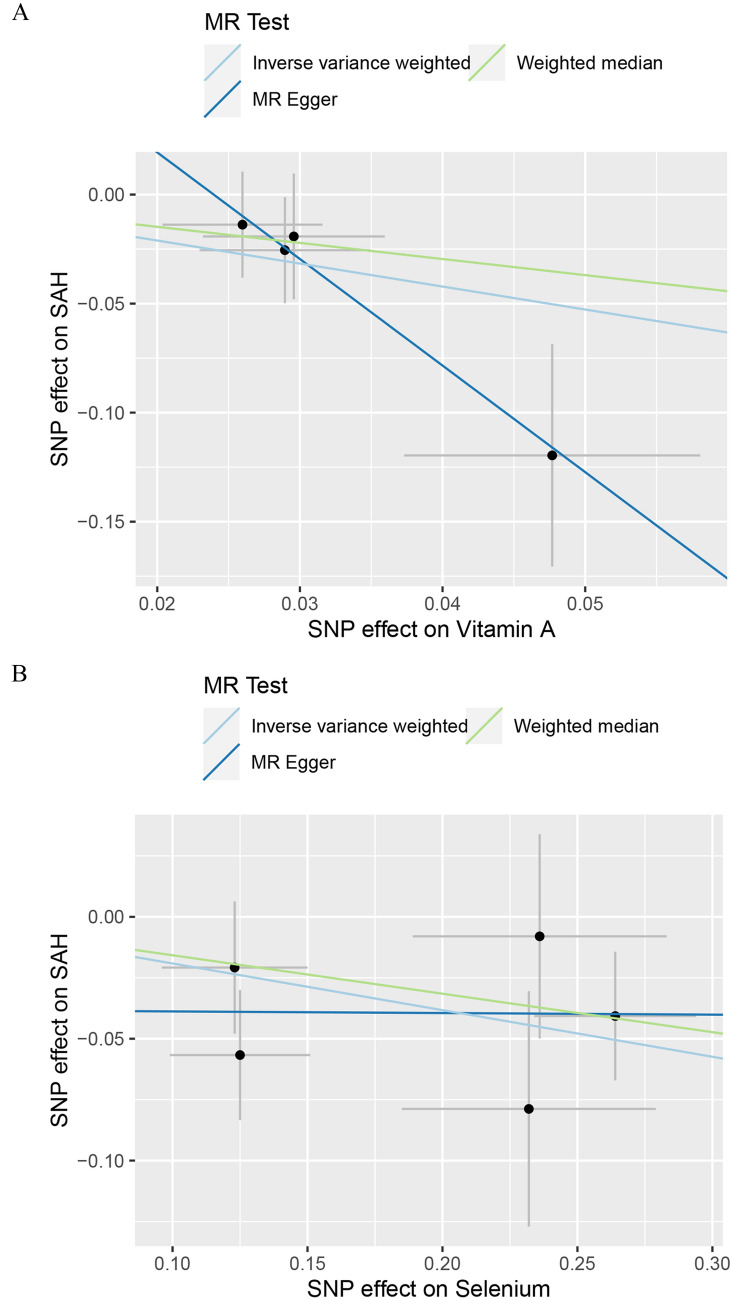
Scatter plots of Mendelian Randomization tests assessing the effect of (**A**) Vitamin A on SAH and (**B**) selenium on SAH. *SAH* subarachnoid hemorrhage

The IVs for antioxidants were summarized in Additional file 2: Table S10. The *F*-statistics of all instrumental variables in this study were greater than 10, which indicated that there was no obvious weak instrumental bias. The primary analysis using IVW showed that genetically determined levels of retinol had a suggestive protective effect on SAH (OR = 0.348, 95% CI 0.138–0.878, *P* = 0.025), and genetically determined levels of selenium had a suggestive protective effect against SAH (OR = 0.826, 95% CI 0.718–0.950, *P* = 0.007) (Fig. 3). However, there was no strong evidence to support a causal relationship between antioxidants and IS or ICH. We continued to examine the effectiveness of other MR methods (Additional file 2: Table S11). However, we did not observe any potent association between retinol or selenium levels and the risk of SAH in any other MR methods (Fig. 4). The relatively low precision of WM and MR-Egger regression might be responsible for this phenomenon, but in general, the direction of each MR method was consistent, indicating that the aforementioned causal relationship was reliable.

Sensitivity analysis of antioxidants on stroke was provided in Additional file 2: Table S12. Cochrane’s Q test demonstrated that vitamin C (ascorbate) was the only exposure with a significant heterogeneity in regards to SAH and ICH. MR-Egger regression did not find horizontal pleiotropy, and no outliers were discovered by MR PRESSO. We also found that, in leave-one-out analysis, the risk estimates for SNPs remained consistent when excluding them individually (Additional file 2: Fig S3, S4).

## Discussion

To the best of our knowledge, this was the first study to investigate the relationship between antioxidants and stroke probability using a cross-sectional and MR design. The results suggested that diet-derived antioxidants might play a role in protecting against stroke.

In NHANES, there was a significant inverse association between CDAI and stroke, whether CDAI was considered as a continuous or categorical variable. And we found that this negative correlation was nonlinear in RCS model, with 3.078 as the inflection point. The risk of stroke decreased by 6.0% for every unit increase in CDAI before the inflection point. However, the association is reversed after the inflection point, although it is not statistically significant. It was possible that vitamin supplements were unnecessary for individuals who consume a balanced diet and were nutritionally adequate. Most notably, a large randomized controlled trial (PHS II) of 14,641 male US physicians found no significant difference in cardiovascular outcomes between the multivitamin group and the placebo group [25]. Furthermore, an excess of supplemental antioxidants in healthy individuals may even be detrimental. According to a meta-analysis study of 78 randomized trials involving 215,900 healthy subjects and 80,807 subjects in stable stages of various diseases, antioxidant supplements were shown to significantly increase mortality (RR = 1.03, 95% CI 1.01–1.05) [26]. These results suggested that, when vitamins and minerals were deficient, antioxidants might have a therapeutic effect to meet individual needs. However, using them in excess might not result in significant additional gains. Furthermore, in the stratified analysis, we observed that the association between CDAI and stroke was particularly prominent among individuals with the highest educational status. This phenomenon might be attributed to differences in health behaviors and healthcare utilization. Those with higher levels of education might have greater access to health-related information regarding the potential advantages of consuming foods rich in antioxidants, and might be more inclined to participate in preventative care and screenings, thereby facilitating the early identification and management of stroke risk factors. Additionally, they might have more resources to procure and consume a nutritious diet, which could potentially reduce their risk of experiencing a stroke.

In MR analysis, the results supported a causal relationship between certain antioxidants and SAH, but not for IS or ICH. Our primary analysis using IVW method revealed suggestive evidence of a protective effect of genetically determined levels of retinol and selenium against SAH. These results were particularly significant given the devastating impact and high mortality rates associated with SAH. Retinol, a form of Vitamin A, is known for its role in supporting vision and immune function [27]. In the context of brain health, it has been studied for its potential neuroprotective effects. Retinol is involved in gene expression, cellular differentiation, and immune function, all of which are critical in maintaining brain health and potentially aiding in recovery post-injury [28]. According to the findings of Marzatico et al., individuals with SAH exhibited notably reduced plasma levels of retinol [29]. This observation suggested that antioxidant capabilities of retinol might be key in mitigating the oxidative stress that could lead to the rupture of intracranial aneurysms, thereby potentially preventing SAH. And Paolo, G et al. implied that diminished levels of retinol might play a role in weakening the function of alpha1-antitrypsin, which was crucial in protease regulation, potentially elevating the risk of cerebral aneurysm rupture and leading to SAH [30]. There was suggesting evidence that elevated levels of circulating selenium might contribute to the development of SAH, which might be due to selenium's protective role in oxidative damage to lipids, as well as its ability to modulate inflammatory and metabolic signaling [31–34]. However, no clinical cohort studies have substantiated the relationship between selenium and SAH. But according to a retrospective study by Yang et al., selenium might help to reduce the incidence of hemorrhagic stroke (OR = 0.68, 95% CI 0.51–0.91) [35]. And Socha et al. found that patients with abdominal aortic aneurysms had lower circulating selenium levels than healthy individuals in a retrospective study [36]. Finally, there was no significant causal relationship detected between genetically determined circulating levels of antioxidants and IS or ICH. In our review and analysis of similar studies, we noted that Martens et al. did not establish a connection between antioxidants and IS [37], whereas Miao et al. revealed a protective effect of genetic predisposition to high circulating γ-tocopherol levels on stroke risk [16]. Analyzing potential reasons for these discrepancies, both studies utilized MEGASTROKE GWAS data for stroke populations but differed in their analytical thresholds. Martens et al. adopted a stringent threshold (P < 5 × 10^–8^), which might have limited the number of instrumental variables, thereby reducing statistical power [37]. In contrast, our study included the largest available GWAS data, utilizing GIGASTROKE for stroke populations, yet our results were negative. This outcome highlights the limitations of older, smaller GWAS datasets for antioxidants and underscores the need for newer, more comprehensive data for stronger conclusions. Furthermore, regarding the relationship between antioxidants and ICH, our findings were also negative. Although Hirvonen et al. found that vitamin C intake was inversely associated with the risk of ICH (RR = 0.39, 95% CI 0.21–0.74) and no relationship between vitamin E and stroke was observed [38]. But Markus et al. pointed out in a meta-analysis that vitamin E supplementation increased the risk for hemorrhagic stroke [39]. These disparities among observational studies and our MR study could stem from unmeasured confounding factors and the issue of reverse causality, highlighting the complexity of determining the impact of antioxidants on stroke subtypes.

## Strengths and limitations

Our study had several advantages, including the combination of cross-sectional study and MR analysis, which reduced the impact of confounding factors and reverse causality. Additionally, by having a large sample size, we were able to reduce selection bias and increase the reliability of our results. Our study also had several limitations. First, the information about strokes in NHANES was self-reported as a history, while the diet-derived antioxidants were estimated during the 24 h period prior to the interview. It was probable that this might result in a recollection bias and consequent misclassification of stroke cases, thus causing a bias in the appraisal of the correlation between dietary antioxidants and the risk of stroke. Second, we excluded a large number of participants with missing stroke and diet-derived intake information data, which could lead to potential selection bias and survivor bias. Thirdly, considering the cross-sectional design, we were unable to obtain follow-up data for stroke outcomes. And it was important to note that the recorded dietary information might not be representative of the diet prior to the outcome of interest, namely, that the diet might have been altered consequent to the stroke. Fourth, compared to other GWAS data, the sample size for ICH was relatively modest. This may explain the lack of a causal relationship. Finally, our findings were based on Europeans and Americans, and ethnic constraints prevented extrapolation of our findings to other populations.

## Conclusions

In this combination of the NHANES cross-sectional study and MR analysis, diet-derived antioxidants might offer some protection against stroke, with the circulating levels of retinol and selenium were beneficial for SAH. However, further exploration is needed to gain a better insight into the mechanisms of antioxidants in preventing stroke, we also hope to explore new prevention strategies and guidelines against stroke to mitigate the human health burden.

### Supplementary Information

**Additional file 1.** ISGC Intracranial Aneurysm Working Group Contributors

**Additional file 2:** Supplementary Figures and Tables. **Figure S1.** Flow chart of eligible National Health and Nutrition Examination Survey (NHANES) participants included in this study. Table S1. Diagnostic criteria of covariates in NHANES. **Figure S2.** The research is based on three hypotheses: (1) The instrumental variable is strongly correlated with diet-derived antioxidants; (2) The instrumental variable is not correlated with the confounding factors; (3) The instrumental variable is not directly related to stroke, and its effect on stroke can only be through diet-derived antioxidants to reflect. **Table S2.** The GWAS summary information of dietary-derived antioxidantsm [1, 2] and stroke [3-5]. **Table S3.** Categorize the CDAI by quartiles. **Table S4.** Association of vitamin A and stroke. **Table S5.** Association of vitamin C and stroke. **Table S6.** Association of vitamin E and stroke. **Table S7.** Association of zinc and stroke. **Table S8.** Association of selenium and stroke. **Table S9.** Association of carotenoid and stroke. **Table S10.** Genetic instrumental variables for dietary-derived antioxidants. **Table S11.** Associations between genetically predicted increase in dietary-derived antioxidants and stroke in Mendelian Randomization analyses. **Table S12.** Sensitivity analysis of antioxidants on stroke. **Figure S3.** Results of leave-one-out sensitivity analysis for Vitamin A on SAH. **Figure S4.** Results of leave-one-out sensitivity analysis for Selenium on SAH.

## Data Availability

The data that support the findings of this study were available from open-source database.

## References

[1] Choi Y, Lee SJ, Spiller W (2020). Causal associations between serum bilirubin levels and decreased stroke risk: a two-sample mendelian randomization study. Arterioscler Thromb Vasc Biol.

[2] Gale CR, Martyn CN, Winter PD (1995). Vitamin C and risk of death from stroke and coronary heart disease in cohort of elderly people. BMJ.

[3] Leppälä JM, Virtamo J, Fogelholm R (1999). Different risk factors for different stroke subtypes: association of blood pressure, cholesterol, and antioxidants. Stroke.

[4] Cheng P, Wang L, Ning S (2018). Vitamin E intake and risk of stroke: a meta-analysis. Br J Nutr.

[5] Mattern L, Chen C, McClure LA (2021). Serum zinc levels and incidence of ischemic stroke: the reasons for geographic and racial differences in stroke study. Stroke.

[6] Loh HC, Lim R, Lee KW (2021). Effects of vitamin E on stroke: a systematic review with meta-analysis and trial sequential analysis. Stroke Vasc Neurol.

[7] Joshipura KJ, Ascherio A, Manson JE (1999). Fruit and vegetable intake in relation to risk of ischemic stroke. JAMA.

[8] Meschia JF, Bushnell C, Boden-Albala B (2014). Guidelines for the primary prevention of stroke: a statement for healthcare professionals from the American Heart Association/American Stroke Association. Stroke.

[9] Lehr HA, Frei B, Arfors KE (1994). Vitamin C prevents cigarette smoke-induced leukocyte aggregation and adhesion to endothelium in vivo. Proc Natl Acad Sci USA.

[10] Patrick L, Uzick M (2001). Cardiovascular disease: C-reactive protein and the inflammatory disease paradigm: HMG-CoA reductase inhibitors, alpha-tocopherol, red yeast rice, and olive oil polyphenols. A review of the literature. Altern Med Rev.

[11] Cobley JN, Fiorello ML, Bailey DM (2018). 13 reasons why the brain is susceptible to oxidative stress. Redox Biol.

[12] Maugeri A, Hruskova J, Jakubik J (2019). Dietary antioxidant intake decreases carotid intima media thickness in women but not in men: a cross-sectional assessment in the Kardiovize study. Free Radic Biol Med.

[13] Sekula P, Del Greco MF, Pattaro C (2016). Mendelian randomization as an approach to assess causality using observational data. J Am Soc Nephrol.

[14] Bowden J, Holmes MV (2019). Meta-analysis and Mendelian randomization: a review. Res Synth Methods.

[15] Gellenbeck KW (1998). Carotenoids: more than just beta-carotene. Asia Pac J Clin Nutr.

[16] Miao R, Li J, Meng C (2022). Diet-derived circulating antioxidants and risk of stroke: a Mendelian randomization study. Oxid Med Cell Longev.

[17] Mishra A, Malik R, Hachiya T (2022). Stroke genetics informs drug discovery and risk prediction across ancestries. Nature.

[18] Woo D, Falcone GJ, Devan WJ (2014). Meta-analysis of genome-wide association studies identifies 1q22 as a susceptibility locus for intracerebral hemorrhage. Am J Hum Genet.

[19] Bakker MK, van der Spek RAA, van Rheenen W (2020). Genome-wide association study of intracranial aneurysms identifies 17 risk loci and genetic overlap with clinical risk factors. Nat Genet.

[20] Burgess S, Butterworth A, Thompson SG (2013). Mendelian randomization analysis with multiple genetic variants using summarized data. Genet Epidemiol.

[21] Bowden J, Davey Smith G, Burgess S (2015). Mendelian randomization with invalid instruments: effect estimation and bias detection through Egger regression. Int J Epidemiol.

[22] Hartwig FP, Davey Smith G, Bowden J (2017). Robust inference in summary data Mendelian randomization via the zero modal pleiotropy assumption. Int J Epidemiol.

[23] Verbanck M, Chen CY, Neale B (2018). Detection of widespread horizontal pleiotropy in causal relationships inferred from Mendelian randomization between complex traits and diseases. Nat Genet.

[24] Larsson SC, Traylor M, Malik R (2017). Modifiable pathways in Alzheimer's disease: Mendelian randomisation analysis. BMJ.

[25] Sesso HD, Christen WG, Bubes V (2012). Multivitamins in the prevention of cardiovascular disease in men: the physicians’ health study II randomized controlled trial. JAMA.

[26] Bjelakovic G, Nikolova D, Gluud LL (2008). Antioxidant supplements for prevention of mortality in healthy participants and patients with various diseases. Cochrane Database Syst Rev.

[27] Sahu B, Maeda A (2016). Retinol dehydrogenases regulate vitamin a metabolism for visual function. Nutrients.

[28] Jakaria M, Belaidi AA, Bush AI (2023). Vitamin A metabolites inhibit ferroptosis. Biomed Pharmacother.

[29] Marzatico F, Gaetani P, Tartara F (1998). Antioxidant status and alpha1-antiproteinase activity in subarachnoid hemorrhage patients. Life Sci.

[30] Paolo G, Paola G, Baena Riccardo YR (1997). Inactivation of alpha1-antiproteinase (alpha1-AT) and changes in antioxidants' plasma levels in subarachnoid hemorrhage. J Neurol Sci.

[31] Hosnedlova B, Kepinska M, Skalickova S (2017). A summary of new findings on the biological effects of selenium in selected animal species-a critical review. Int J Mol Sci.

[32] Amani H, Habibey R, Shokri F (2019). Selenium nanoparticles for targeted stroke therapy through modulation of inflammatory and metabolic signaling. Sci Rep.

[33] Alim I, Caulfield JT, Chen Y (2019). Selenium drives a transcriptional adaptive program to block ferroptosis and treat stroke. Cell.

[34] Czekajło A (2019). Role of diet-related factors in cerebral aneurysm formation and rupture. Rocz Panstw Zakl Hig.

[35] Xiao Y, Yuan Y, Liu Y (2019). Circulating multiple metals and incident stroke in chinese adults. Stroke.

[36] Socha K, Borawska MH, Gacko M (2011). Diet and the content of selenium and lead in patients with abdominal aortic aneurysm. Vasa.

[37] Martens LG, Luo J, van Willems Dijk K (2021). Diet-derived antioxidants do not decrease risk of ischemic stroke: a mendelian randomization study in 1 million people. J Am Heart Assoc.

[38] Hirvonen T, Virtamo J, Korhonen P (2000). Intake of flavonoids, carotenoids, vitamins C and E, and risk of stroke in male smokers. Stroke.

[39] Schürks M, Glynn RJ, Rist PM (2010). Effects of vitamin E on stroke subtypes: meta-analysis of randomised controlled trials. BMJ.

