# Supplementary material for: Antioxidants and the risk of stroke: results from NHANES and two-sample Mendelian randomization study
Source: Eur J Med Res. 2024 Jan 12;29:50. doi: 10.1186/s40001-024-01646-5 (PMC10785483; doi:10.1186/s40001-024-01646-5)
Supplement: Supplementary file 1 — Additional file 1. ISGC Intracranial Aneurysm Working Group Contributors [file 40001_2024_1646_MOESM1_ESM.docx]

**International Stroke Genetics Consortium (ISGC) Intracranial Aneurysm Working Group**

Mark K. Bakker^1^ , Romain Bourcier^2,3^, Robin G. Walters^4,5^, Rainer Malik^6^, Martin Dichgans^6,7,8^, Muralidharan Sargurupremraj^9,10^, Turgut Tatlisumak^11^, Stéphanie Debette^9,10^, Gabriel J.E. Rinkel^1^ , Bradford B. Worrall^12^, Joanna Pera^13^, Agnieszka Slowik^13^, Joseph P. Broderick^14^, David J. Werring^15^, Daniel Woo^14^, Philippe Bijlenga^16^, Yoichiro Kamatani^17^, Ynte M. Ruigrok1

1 Department of Neurology and Neurosurgery, University Medical Center Utrecht Brain Center, Utrecht University, Utrecht, The Netherlands.

2 Université de Nantes, CHU Nantes, INSERM, CNRS, l'institut du thorax, Nantes, France.

3 CHU Nantes, Department of Neuroradiology, Nantes, France.

4 Clinical Trial Service Unit and Epidemiological Studies Unit, Nuffield Department of Population Health, University of Oxford, Oxford, U.K.

5 Medical Research Council Population Health Research Unit, University of Oxford, Oxford, U.K.

6 Institute for Stroke and Dementia Research, University Hospital, Ludwig-Maximilians-University, Munich.

7 Munich Cluster for Systems Neurology (SyNergy), Munich, Germany.

8 Deutsches Zentrum für Neurodegenerative Erkrankungen (DZNE), Munich, Germany.

9 INSERM U1219 Bordeaux Population Health Research Center, University of Bordeaux, Bordeaux, France.

10Department of Neurology, Institute for Neurodegenerative Disease, Bordeaux University Hospital, Bordeaux, France.

11Department of Clinical Neuroscience at Institute of Neuroscience and Physiology, University of Gothenburg, Sweden.

12Departments of Neurology and Public Health Sciences, University of Virginia School of Medicine, Charlottesville, VA, USA.

13Department of Neurology, Faculty of Medicine, Jagiellonian University Medical College, ul. Botaniczna 3, 31-503, Krakow, Poland.

14University of Cincinnati College of Medicine, Cincinnati, OH, USA.

15Stroke Research Centre, University College London Queen Square Institute of Neurology, London, UK.

16Neurosurgery Division, Department of Clinical Neurosciences, Faculty of Medicine, Geneva University Hospitals, Geneva, Switzerland. 17Graduate School of Frontier Sciences, The University of Tokyo, Tokyo, Japan
