# Supplementary material for: Antioxidants and the risk of stroke: results from NHANES and two-sample Mendelian randomization study
Source: Eur J Med Res. 2024 Jan 12;29:50. doi: 10.1186/s40001-024-01646-5 (PMC10785483; doi:10.1186/s40001-024-01646-5)
Supplement: Supplementary file 2 — Additional file 2: Supplementary Figures and Tables. Figure S1. Flow chart of eligible National Health and Nutrition Examination Survey (NHANES) participants included in this study. Table S1. Diagnostic criteria of covariates in NHANES. Figure S2. The research is based on three hypotheses: (1) The instrumental variable is strongly correlated with diet-derived antioxidants; (2) The instrumental variable is not correlated with the confounding factors; (3) The instrumental variable is not directly related to stroke, and its effect on stroke can only be through diet-derived antioxidants to reflect. Table S2. The GWAS summary information of dietary-derived antioxidantsm [1, 2] and stroke [3-5]. Table S3. Categorize the CDAI by quartiles. Table S4. Association of vitamin A and stroke. Table S5. Association of vitamin C and stroke. Table S6. Association of vitamin E and stroke. Table S7. Association of zinc and stroke. Table S8. Association of selenium and stroke. Table S9. Association of carotenoid and stroke. Table S10. Genetic instrumental variables for dietary-derived antioxidants. Table S11. Associations between genetically predicted increase in dietary-derived antioxidants and stroke in Mendelian Randomization analyses. Table S12. Sensitivity analysis of antioxidants on stroke. Figure S3. Results of leave-one-out sensitivity analysis for Vitamin A on SAH. Figure S4. Results of leave-one-out sensitivity analysis for Selenium on SAH. [file 40001_2024_1646_MOESM2_ESM.docx]

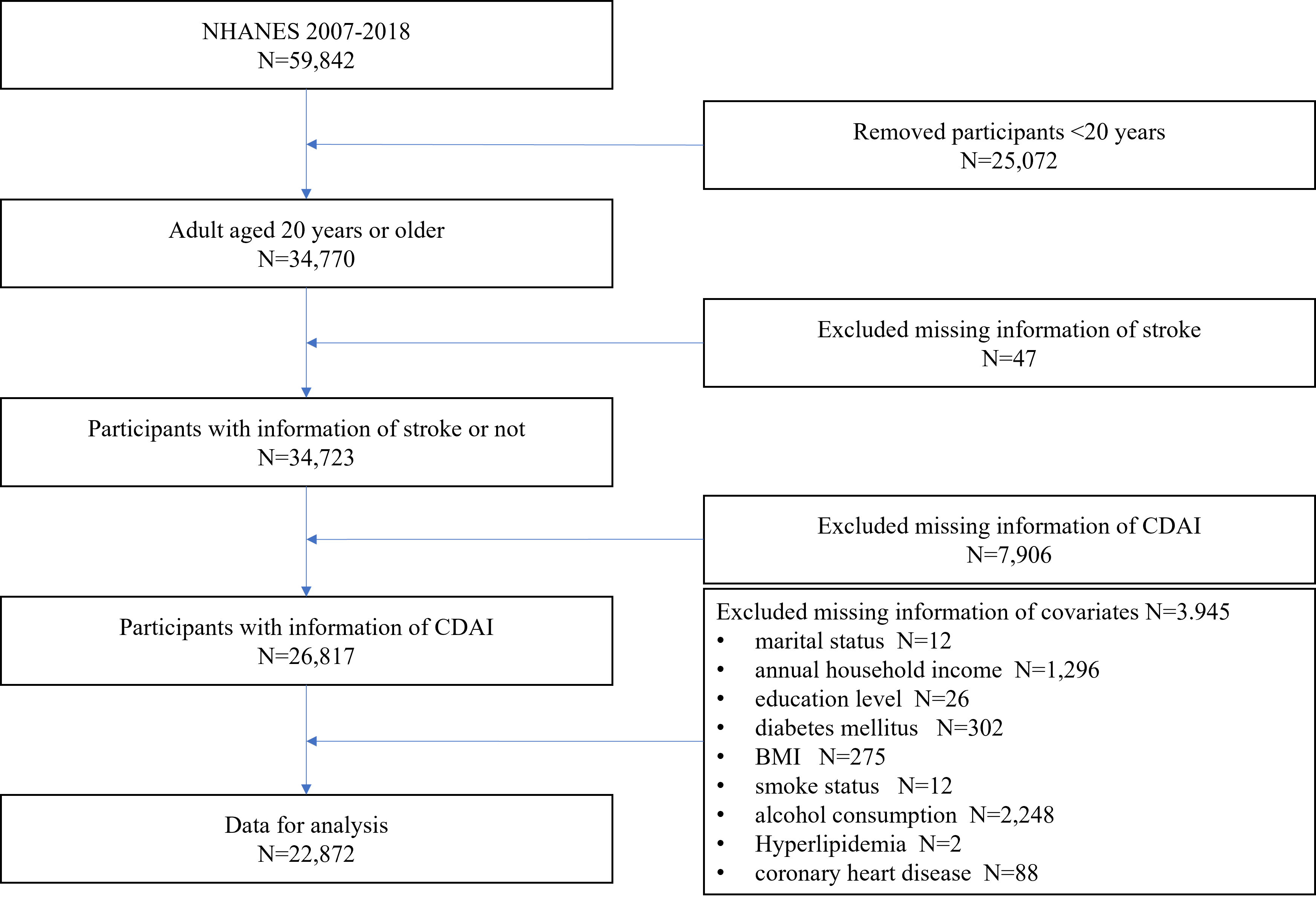


**Figure S1** Flow chart of eligible National Health and Nutrition Examination Survey (NHANES) participants included in this study.

**Table S1** Diagnostic criteria of covariates in NHANES

| Variable | Diagnostic criteria |
| --- | --- |
| Age | Age in years of the participant at the time of screening. Individuals 80 and over are topcoded at 80 years of age. This variable was subdivided into two groups: those aged 70 or above, and those aged below 70. |
| Gender | Gender of the participant. This variable was subdivided into two groups: female and male |
| Race | Recode of reported race and Hispanic origin information. This variable was subdivided into four groups: Mexican American, non-Hispanic White, non-Hispanic Black and others. |
| Marital status: | This variable was subdivided into two groups: married/partnered, separated/widowed and single. |
| Annual household income | Total household income (reported as a range value in dollars). This variable was subdivided into two groups: those earning more than or equal to $20,000 and those earning less than $20,000. |
| Education level | Response of “what is the highest grade or level of school ^[1]^ completed or the highest degree {you have/s/he has} received?” This variable was subdivided into two groups: senior high schools or GED, junior middle schools or below, and colleges or above. |
| Body mass index (BMI) | kg/m2. This variable was subdivided into three groups: those with a BMI less than 25, those with a BMI between 25 and 30, and those with a BMI greater than or equal to 30. |
| Smoke status: | Response to “{Have you/Has SP} smoked at least 100 cigarettes in {your/his/her} entire life?”. This variable was subdivided into three groups: yes and no. |
| Alcohol consumption: | Response to “In any one year, {have you/has SP} had at least 12 drinks of any type of alcoholic beverage? By a drink, I mean a 12 oz. beer, a 5 oz. glass of wine, or a one and a half ounces of liquor.” This variable was subdivided into three groups: yes and no. |
| Diabetes mellitus: | The diagnostic criteria for diabetes are: the doctor told you have diabetes or glycohemoglobin HbA1c(%) greater than 6.5 or use of diabetes medication or insulin. This variable was subdivided into three groups: yes and no. |
| Hypertension: | The diagnostic criteria for hypertension are: the doctor told you have hypertension or use of medication for hypertension or systolic more than 140 or diastolic more than 90. Average blood pressure was calculated by the following protocol: The diastolic reading with zero is not used to calculate the diastolic average; If all diastolic readings were zero, then the average would be zero; If only one blood pressure reading was obtained, that reading is the average; If there is more than one blood pressure reading, the first reading is always excluded from the average. This variable was subdivided into three groups: yes and no. |
| Hyperlipidemia | Hyperlipidemia refers to an elevation of triglycerides or cholesterol in the blood, or the use of lipid-lowering drugs. Hypertriglyceridemia is defined as triglycerides levels greater than or equal to 150mg/dl, while hypercholesterolemia is defined as total cholesterol levels greater than or equal to 200mg/dl, or low-density lipoprotein levels greater than or equal to 130mg/dl. High-density lipoprotein levels are considered low if they are less than 40mg/dl for men or less than 50mg/dl for women. This variable was subdivided into three groups: yes and no. |
| Coronary heart disease | Response to “Has a doctor or other health professional ever told {you/SP}T that {you/s/he} . . .had coronary heart disease?” This variable was subdivided into three groups: yes and no. |
| Physical activity | Physical activity was measured in MET minutes of moderate to vigorous physical activity per week, and participants were categorized as inactive, low (more than 0 but less than 600 MET-minutes of moderate-intensity physical activity), and high (more than 600 MET-minutes of moderate-intensity physical activity) |


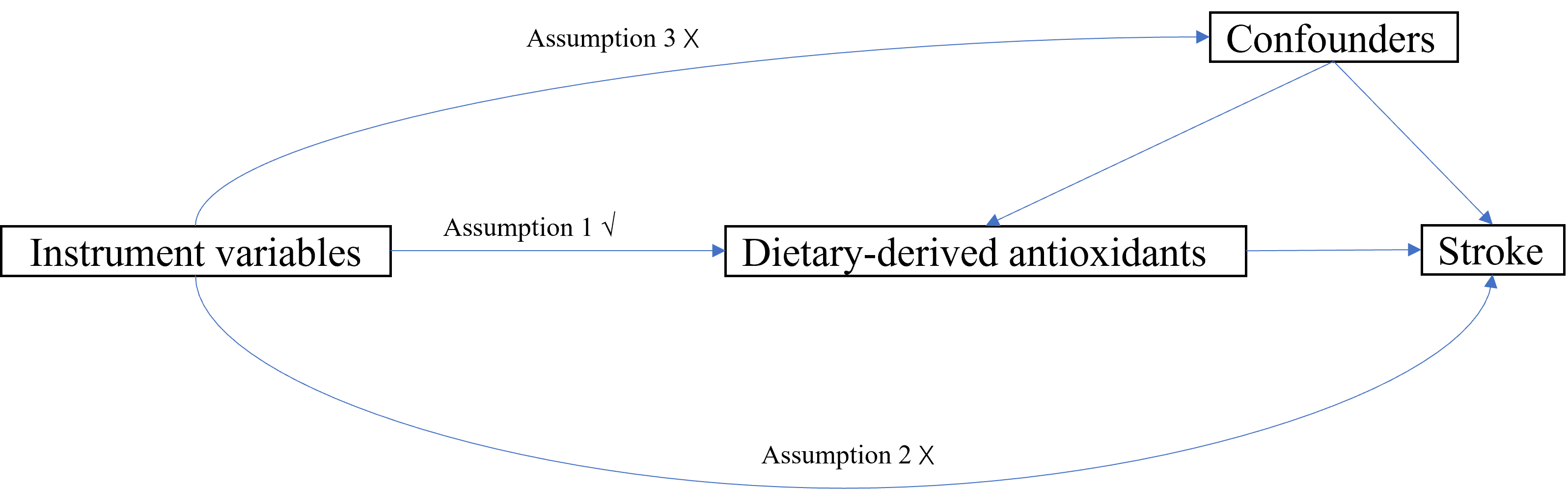


**Figure S2** The research is based on three hypotheses: (1) The instrumental variable is strongly correlated with diet-derived antioxidants; (2) The instrumental variable is not correlated with the confounding factors; (3) The instrumental variable is not directly related to stroke, and its effect on stroke can only be through diet-derived antioxidants to reflect.

**Table S2** The GWAS summary information of dietary-derived antioxidants^[1, 2]^ and stroke^[3-5]^

| Trait | Build | Year | Author | Sample size | PMID | nsnp | Unit | Population |
| --- | --- | --- | --- | --- | --- | --- | --- | --- |
| Vitamin A (retinol) | HG19/GRCh37 | 2018 | Ben Elsworth | total 62,991 | Public | 9,851,867 | SD | European |
| Vitamin C (ascorbate) | HG19/GRCh37 | 2018 | Ben Elsworth | total 64,979 | NA | 9,851,867 | SD | European |
| Vitamin E (α-tocopherol) | HG19/GRCh37 | 2014 | Shin | total 7,725 | 24816252 | 2,545,636 | log10 units | European |
| Vitamin E (γ-tocopherol) | HG19/GRCh37 | 2014 | Shin | total 6,226 | 24816252 | 2,544,979 | log10 units | European |
| Zinc | HG19/GRCh37 | 2013 | Evans | total 2,603 | 23720494 | 2,543,610 | SD | European |
| Selenium | HG19/GRCh37 | 2013 | Evans | total 2,874 | 23720494 | 2,451,527 | SD | European |
| Carotene | HG19/GRCh37 | 2018 | Ben Elsworth | total 64,979 | Public | 9,851,867 | SD | European |
| Ischemic stroke | HG19/GRCh37 | 2022 | Mishra | 110,182 cases / 1,503,898 controls | 36376532 | 7,537,579 | NA | European |
| Nontraumatic intracranial hemorrhage | HG19/GRCh37 | 2014 | Woo Daniel | 1514 cases / 1481 controls | 24656865 | 16,380,408 | NA | European |
| Subarachnoid hemorrhage | HG19/GRCh37 | 2020 | Bakker MK | 5140 cases / 71,934 controls | 33199917 | 3,533,714 | NA | European |

**Table S3** Categorize the CDAI by quartiles

|  | Range | Median | Frequent | Percentage |
| --- | --- | --- | --- | --- |
| Lowest quartiles | [-7.379,-2.534] | -3.628 | 6614 | 25.02% |
| Q2 | (-2.534,-0.647] | -1.599 | 6607 | 25% |
| Q3 | (-0.647,1.732] | 0.418 | 6604 | 24.98% |
| Q4 | (1.732,71.998] | 3.893 | 6608 | 25% |

**Table S4** Association of vitamin A and stroke.

|  | Crude model^a^ | | Model1^b^ | | Model 2^c^ | |
| --- | --- | --- | --- | --- | --- | --- |
|  | OR (95%CI) | *P* | OR (95%CI) | *P* | OR (95%CI) | *P* |
| CDAI | 1.000(1.000,1.000) | 0.165 | 1.000(1.000,1.000) | 0.092 | 1.000(1.000,1.000) | 0.513 |
| Lowest quartiles | ref | | | | | |
| Q2 | 0.886(0.718,1.094) | 0.258 | 0.807(0.648,1.005) | 0.055 | 0.851(0.676,1.070) | 0.164 |
| Q3 | 0.737(0.579,0.938) | 0.014 | 0.655(0.505,0.850) | 0.002 | 0.721(0.546,0.951) | 0.021 |
| Q4 | 0.738(0.582,0.935) | 0.013 | 0.662(0.517,0.846) | 0.001 | 0.828(0.649,1.058) | 0.130 |
| *P* for trend^d^ |  | 0.016 |  | 0.003 |  | 0.19 |

**Table S5** Association of vitamin C and stroke.

|  | Crude model^a^ | | Model1^b^ | | Model 2^c^ | |
| --- | --- | --- | --- | --- | --- | --- |
|  | OR (95%CI) | *P* | OR (95%CI) | *P* | OR (95%CI) | *P* |
| CDAI | 0.999(0.998,1.001) | 0.343 | 0.999(0.998,1.001) | 0.297 | 1.000(0.999,1.002) | 0.848 |
| Lowest quartiles | ref | | | | | |
| Q2 | 0.902(0.704,1.156) | 0.410 | 0.861(0.671,1.106) | 0.238 | 0.965(0.743,1.253) | 0.787 |
| Q3 | 0.618(0.489,0.782) | <0.0001 | 0.534(0.422,0.676) | <0.0001 | 0.641(0.507,0.810) | <0.001 |
| Q4 | 0.769(0.614,0.964) | 0.023 | 0.698(0.554,0.880) | 0.003 | 0.914(0.712,1.174) | 0.477 |
| *P* for trend^d^ |  | 0.019 |  | 0.003 |  | 0.361 |

**Table S6** Association of vitamin E and stroke.

|  | Crude model^a^ | | Model1^b^ | | Model 2^c^ | |
| --- | --- | --- | --- | --- | --- | --- |
|  | OR (95%CI) | *P* | OR (95%CI) | *P* | OR (95%CI) | *P* |
| CDAI | 0.947(0.929,0.965) | <0.0001 | 0.956(0.938,0.974) | <0.0001 | 0.973(0.957,0.990) | 0.002 |
| Lowest quartiles | ref | | | | | |
| Q2 | 0.897(0.717,1.123) | 0.338 | 0.925(0.732,1.169) | 0.510 | 1.095(0.857,1.400) | 0.463 |
| Q3 | 0.681(0.548,0.848) | <0.001 | 0.747(0.598,0.933) | 0.011 | 0.942(0.759,1.170) | 0.585 |
| Q4 | 0.516(0.404,0.658) | <0.0001 | 0.598(0.463,0.774) | <0.001 | 0.796(0.613,1.034) | 0.087 |
| *P* for trend^d^ |  | <0.0001 |  | <0.0001 |  | 0.024 |

**Table S7** Association of zinc and stroke.

|  | Crude model^a^ | | Model1^b^ | | Model 2^c^ | |
| --- | --- | --- | --- | --- | --- | --- |
|  | OR (95%CI) | *P* | OR (95%CI) | *P* | OR (95%CI) | *P* |
| CDAI | 0.953(0.937,0.969) | <0.0001 | 0.965(0.950,0.980) | <0.0001 | 0.978(0.963,0.993) | 0.005 |
| Lowest quartiles | ref | | | | | |
| Q2 | 0.776(0.633,0.952) | 0.016 | 0.820(0.663,1.013) | 0.066 | 0.888(0.716,1.100) | 0.272 |
| Q3 | 0.605(0.473,0.773) | <0.0001 | 0.691(0.542,0.880) | 0.003 | 0.800(0.621,1.031) | 0.084 |
| Q4 | 0.480(0.371,0.622) | <0.0001 | 0.589(0.453,0.765) | <0.001 | 0.719(0.548,0.944) | 0.018 |
| *P* for trend^d^ |  | <0.0001 |  | <0.0001 |  | 0.007 |

**Table S8** Association of selenium and stroke.

|  | Crude model^a^ | | Model1^b^ | | Model 2^c^ | |
| --- | --- | --- | --- | --- | --- | --- |
|  | OR (95%CI) | P | OR (95%CI) | P | OR (95%CI) | P |
| CDAI | 0.994(0.992,0.996) | <0.0001 | 0.995(0.993,0.997) | <0.0001 | 0.997(0.995,0.999) | 0.001 |
| Lowest quartiles | ref | | | | | |
| Q2 | 0.680(0.557,0.829) | <0.001 | 0.730(0.595,0.896) | 0.003 | 0.783(0.631,0.971) | 0.027 |
| Q3 | 0.477(0.376,0.605) | <0.0001 | 0.553(0.436,0.702) | <0.0001 | 0.621(0.490,0.789) | <0.001 |
| Q4 | 0.380(0.282,0.512) | <0.0001 | 0.491(0.358,0.674) | <0.0001 | 0.590(0.430,0.809) | 0.001 |
| *P* for trend^d^ |  | <0.0001 |  | <0.0001 |  | <0.001 |

**Table S9** Association of carotenoid and stroke.

|  | Crude model^a^ | | Model1^b^ | | Model 2^c^ | |
| --- | --- | --- | --- | --- | --- | --- |
|  | OR (95%CI) | *P* | OR (95%CI) | *P* | OR (95%CI) | *P* |
| CDAI | 1.000(1.000,1.000) | 0.214 | 1.000(1.000,1.000) | 0.448 | 1.000(1.000,1.000) | 0.681 |
| Lowest quartiles | ref | | | | | |
| Q1 | ref |  | ref |  | ref |  |
| Q2 | 0.697(0.567,0.856) | <0.001 | 0.752(0.616,0.918) | 0.006 | 0.822(0.668,1.012) | 0.064 |
| Q3 | 0.568(0.455,0.708) | <0.0001 | 0.599(0.478,0.751) | <0.0001 | 0.686(0.543,0.866) | 0.002 |
| *P* for trend^d^ |  | 0.029 |  | 0.115 |  | 0.768 |

Table S4-S9:

^a^Crude model: no covariates were adjusted.

^b^Model 1: age, gender, and race were adjusted.

^c^Model 2: age, gender, race, education level, marital status, annual household income, smoking status, alcohol consumption, BMI, diabetes mellitus, hypertension, hyperlipidemia, coronary artery disease and physical activity were adjusted.

^d^Test for trend based on variable containing median value for each quintile.

**Table S10** Genetic instrumental variables for dietary-derived antioxidants

| Trait | Sequence | Chr | SNP | Effect allele | Other allele | Eaf | Beta | SE | *P* |
| --- | --- | --- | --- | --- | --- | --- | --- | --- | --- |
| Vitamin A (retinol) | 1 | 1 | rs12119164 | G | A | 0.742193 | 0.029574 | 0.00637063 | 3.40E-06 |
|  | 2 | 1 | rs692790 | C | T | 0.874331 | 0.040415 | 0.00838208 | 1.40E-06 |
|  | 3 | 2 | rs74977546 | A | G | 0.052896 | -0.06351 | 0.0126784 | 5.50E-07 |
|  | 4 | 3 | rs149577802 | T | C | 0.015116 | -0.1088 | 0.0234115 | 3.40E-06 |
|  | 5 | 10 | rs3213829 | G | T | 0.546482 | 0.025976 | 0.00560984 | 3.60E-06 |
|  | 6 | 14 | rs117669768 | A | G | 0.038236 | 0.078955 | 0.0147682 | 9.00E-08 |
|  | 7 | 16 | rs2126371 | T | C | 0.317531 | -0.02895 | 0.00597696 | 1.30E-06 |
|  | 8 | 16 | rs117219913 | C | T | 0.07834 | 0.047686 | 0.0103813 | 4.40E-06 |
|  | 9 | 20 | rs909570 | A | G | 0.938952 | -0.05314 | 0.0115717 | 4.40E-06 |
| Vitamin E (α-tocopherol) | 1 | 7 | rs1404410 | G | C | 0.2147 | 0.0236 | 0.0052 | 4.57E-06 |
|  | 2 | 7 | rs10245705 | T | C | 0.0181 | -0.0663 | 0.0127 | 1.95E-07 |
|  | 3 | 9 | rs11145330 | C | A | 0.1092 | -0.0324 | 0.0068 | 1.95E-06 |
|  | 4 | 18 | rs7238006 | C | T | 0.0738 | -0.0281 | 0.0057 | 6.77E-07 |
|  | 5 | 22 | rs2074731 | A | C | 0.1654 | -0.0184 | 0.0039 | 2.31E-06 |
| Vitamin E (γ-tocopherol) | 1 | 5 | rs10077932 | T | C | 0.1376 | -0.0402 | 0.0087 | 4.08E-06 |
|  | 2 | 9 | rs7038957 | C | T | 0.1688 | 0.0287 | 0.0062 | 3.86E-06 |
|  | 3 | 15 | rs7350776 | G | C | 0.303 | -0.0239 | 0.0052 | 3.86E-06 |
|  | 4 | 15 | rs261301 | C | T | 0.8686 | -0.0323 | 0.0068 | 2.06E-06 |
|  | 5 | 17 | rs1013104 | T | C | 0.4354 | -0.0207 | 0.0045 | 3.83E-06 |
|  | 6 | 19 | rs1060467 | G | A | 0.4097 | -0.0233 | 0.0045 | 2.61E-07 |
|  | 7 | 22 | rs5994305 | G | A | 0.1682 | -0.0307 | 0.0062 | 7.15E-07 |
| Vitamin C (ascorbate) | 1 | 3 | rs7626478 | A | G | 0.7203 | 0.0280 | 0.0061 | 4.50E-06 |
|  | 2 | 3 | rs114598078 | T | C | 0.0423 | 0.0656 | 0.0138 | 1.90E-06 |
|  | 3 | 3 | rs4481190 | C | A | 0.3510 | -0.0306 | 0.0057 | 9.60E-08 |
|  | 4 | 7 | rs74978963 | T | C | 0.0087 | 0.1508 | 0.0310 | 1.20E-06 |
|  | 5 | 10 | rs61868302 | T | C | 0.0607 | -0.0571 | 0.0118 | 1.40E-06 |
|  | 6 | 12 | rs17482258 | T | C | 0.0991 | 0.0428 | 0.0093 | 3.70E-06 |
|  | 7 | 12 | rs2018201 | G | T | 0.0266 | -0.0808 | 0.0172 | 2.50E-06 |
|  | 8 | 13 | rs9540734 | A | G | 0.4775 | -0.0259 | 0.0055 | 2.30E-06 |
|  | 9 | 15 | rs4238567 | C | T | 0.5220 | 0.0253 | 0.0055 | 4.30E-06 |
|  | 10 | 17 | rs11650824 | A | T | 0.0351 | 0.0795 | 0.0159 | 5.60E-07 |
|  | 11 | 22 | rs1883993 | A | G | 0.0954 | 0.0450 | 0.0094 | 1.50E-06 |
| Zinc | 1 | 2 | rs10931753 | C | G | NA | -0.129 | 0.028 | 4.94E-06 |
|  | 2 | 4 | rs4333127 | A | G | NA | 0.218 | 0.047 | 3.00E-06 |
|  | 3 | 7 | rs11763353 | G | A | NA | -0.192 | 0.039 | 6.90E-07 |
|  | 4 | 8 | rs1532423 | G | A | NA | -0.178 | 0.026 | 6.40E-12 |
|  | 5 | 11 | rs11232535 | C | T | NA | 0.325 | 0.065 | 6.73E-07 |
|  | 6 | 14 | rs7148590 | A | G | NA | -0.14 | 0.026 | 1.37E-07 |
|  | 7 | 14 | rs10484100 | G | A | NA | -0.209 | 0.045 | 3.30E-06 |
|  | 8 | 15 | rs2120019 | C | T | NA | -0.287 | 0.033 | 1.55E-18 |
| Selenium | 1 | 2 | rs3770549 | T | A | NA | 0.187 | 0.04 | 2.39E-06 |
|  | 2 | 4 | rs6823178 | A | G | NA | -0.125 | 0.026 | 2.32E-06 |
|  | 3 | 5 | rs921943 | T | C | NA | 0.264 | 0.03 | 1.43E-18 |
|  | 4 | 5 | rs11948804 | T | C | NA | 0.236 | 0.047 | 3.90E-07 |
|  | 5 | 16 | rs2631524 | G | A | NA | 0.15 | 0.032 | 1.98E-06 |
|  | 6 | 17 | rs12951643 | A | G | NA | -0.232 | 0.047 | 8.01E-07 |
|  | 7 | 22 | rs9609603 | C | T | NA | 0.123 | 0.027 | 4.00E-06 |
| Carotene | 1 | 1 | rs1936052 | T | C | 0.155936 | -0.0361185 | 0.00766833 | 2.50E-06 |
|  | 2 | 1 | rs6660246 | C | A | 0.450266 | -0.0266889 | 0.00553072 | 1.40E-06 |
|  | 3 | 1 | rs12126792 | G | A | 0.011509 | -0.134712 | 0.0280049 | 1.50E-06 |
|  | 4 | 4 | rs77547747 | C | T | 0.056418 | -0.055803 | 0.0118339 | 2.40E-06 |
|  | 5 | 5 | rs6596473 | C | G | 0.299056 | 0.0276423 | 0.00597629 | 3.70E-06 |
|  | 6 | 6 | rs62417408 | G | A | 0.037772 | -0.0691153 | 0.0146553 | 2.40E-06 |
|  | 7 | 8 | rs16898247 | A | G | 0.018968 | -0.107091 | 0.0200309 | 9.00E-08 |
|  | 8 | 9 | rs13295574 | A | G | 0.303383 | -0.0276722 | 0.00598182 | 3.70E-06 |
|  | 9 | 10 | rs3829931 | A | T | 0.973235 | 0.0830829 | 0.0177899 | 3.00E-06 |
|  | 10 | 10 | rs17800766 | C | T | 0.011673 | -0.121685 | 0.0254964 | 1.80E-06 |
|  | 11 | 10 | rs2998143 | G | A | 0.603581 | -0.0278722 | 0.00584016 | 1.80E-06 |
|  | 12 | 13 | rs4771831 | A | G | 0.351886 | -0.0265212 | 0.00576207 | 4.20E-06 |
|  | 13 | 16 | rs116995905 | T | C | 0.009812 | -0.132301 | 0.0288061 | 4.40E-06 |
|  | 14 | 19 | rs366337 | G | A | 0.936764 | 0.0543804 | 0.0112221 | 1.30E-06 |
|  | 15 | 22 | rs117731008 | A | G | 0.016818 | 0.0977133 | 0.0212277 | 4.20E-06 |
|  | 16 | 22 | rs5760695 | C | T | 0.086598 | 0.0468481 | 0.0100556 | 3.20E-06 |

**Table S11** Associations between genetically predicted increase in dietary-derived antioxidants and stroke in Mendelian Randomization analyses

| Outcome | Exposure | Method | nsnp | Beta | SE | *P* | Lower CI | Upper CI | OR | Lower CI | Upper CI |
| --- | --- | --- | --- | --- | --- | --- | --- | --- | --- | --- | --- |
| Vitamin A (retinol) | IS | Inverse variance weighted | 7 | -0.059 | 0.139 | 0.675 | -0.332 | 0.215 | 0.943 | 0.718 | 1.240 |
| Vitamin A (retinol) | IS | MR Egger | 7 | -0.108 | 0.372 | 0.784 | -0.838 | 0.622 | 0.898 | 0.433 | 1.863 |
| Vitamin A (retinol) | IS | Weighted median | 7 | -0.113 | 0.143 | 0.430 | -0.394 | 0.168 | 0.893 | 0.674 | 1.183 |
| Vitamin C (ascorbate) | IS | Inverse variance weighted | 9 | 0.069 | 0.095 | 0.465 | -0.117 | 0.255 | 1.072 | 0.890 | 1.291 |
| Vitamin C (ascorbate) | IS | MR Egger | 9 | 0.260 | 0.291 | 0.401 | -0.310 | 0.831 | 1.297 | 0.733 | 2.296 |
| Vitamin C (ascorbate) | IS | Weighted median | 9 | 0.091 | 0.124 | 0.464 | -0.152 | 0.334 | 1.095 | 0.859 | 1.396 |
| Vitamin E (α-tocopherol) | IS | Inverse variance weighted | 4 | -0.026 | 0.200 | 0.895 | -0.418 | 0.366 | 0.974 | 0.658 | 1.442 |
| Vitamin E (α-tocopherol) | IS | MR Egger | 4 | -0.488 | 0.552 | 0.470 | -1.569 | 0.594 | 0.614 | 0.208 | 1.811 |
| Vitamin E (α-tocopherol) | IS | Weighted median | 4 | -0.075 | 0.232 | 0.745 | -0.530 | 0.379 | 0.927 | 0.589 | 1.461 |
| Vitamin E (γ-tocopherol) | IS | Inverse variance weighted | 6 | -0.024 | 0.145 | 0.870 | -0.308 | 0.261 | 0.977 | 0.735 | 1.298 |
| Vitamin E (γ-tocopherol) | IS | MR Egger | 6 | 0.732 | 0.607 | 0.295 | -0.458 | 1.922 | 2.079 | 0.632 | 6.838 |
| Vitamin E (γ-tocopherol) | IS | Weighted median | 6 | -0.051 | 0.162 | 0.754 | -0.368 | 0.266 | 0.951 | 0.692 | 1.305 |
| Zinc | IS | Inverse variance weighted | 7 | 0.007 | 0.017 | 0.671 | -0.026 | 0.040 | 1.007 | 0.974 | 1.041 |
| Zinc | IS | MR Egger | 7 | -0.072 | 0.063 | 0.305 | -0.195 | 0.052 | 0.931 | 0.823 | 1.053 |
| Zinc | IS | Weighted median | 7 | -0.004 | 0.022 | 0.867 | -0.046 | 0.039 | 0.996 | 0.955 | 1.040 |
| Selenium | IS | Inverse variance weighted | 6 | 0.016 | 0.019 | 0.412 | -0.022 | 0.053 | 1.016 | 0.978 | 1.055 |
| Selenium | IS | MR Egger | 6 | 0.028 | 0.059 | 0.663 | -0.088 | 0.144 | 1.028 | 0.915 | 1.155 |
| Selenium | IS | Weighted median | 6 | 0.031 | 0.022 | 0.160 | -0.012 | 0.075 | 1.032 | 0.988 | 1.078 |
| Carotene | IS | Inverse variance weighted | 11 | -0.011 | 0.085 | 0.898 | -0.177 | 0.156 | 0.989 | 0.837 | 1.168 |
| Carotene | IS | MR Egger | 11 | -0.078 | 0.201 | 0.706 | -0.472 | 0.316 | 0.925 | 0.624 | 1.371 |
| Carotene | IS | Weighted median | 11 | 0.026 | 0.115 | 0.818 | -0.200 | 0.253 | 1.027 | 0.819 | 1.287 |
| Vitamin A (retinol) | ICH | Inverse variance weighted | 5 | -0.093 | 0.864 | 0.915 | -1.786 | 1.600 | 0.912 | 0.168 | 4.955 |
| Vitamin A (retinol) | ICH | MR Egger | 5 | 1.606 | 3.259 | 0.656 | -4.782 | 7.993 | 4.981 | 0.008 | 2961.005 |
| Vitamin A (retinol) | ICH | Weighted median | 5 | 0.295 | 1.154 | 0.798 | -1.966 | 2.556 | 1.343 | 0.140 | 12.888 |
| Vitamin C (ascorbate) | ICH | Inverse variance weighted | 6 | 1.512 | 0.872 | 0.083 | -0.198 | 3.221 | 4.534 | 0.820 | 25.054 |
| Vitamin C (ascorbate) | ICH | MR Egger | 6 | 4.892 | 2.258 | 0.096 | 0.467 | 9.318 | 133.244 | 1.595 | 11132.038 |
| Vitamin C (ascorbate) | ICH | Weighted median | 6 | 0.839 | 1.120 | 0.454 | -1.358 | 3.035 | 2.313 | 0.257 | 20.793 |
| Vitamin E (α-tocopherol) | ICH | Inverse variance weighted^a^ | 4 | 1.215 | 1.503 | 0.419 | -1.731 | 4.162 | 3.371 | 0.177 | 64.168 |
| Vitamin E (α-tocopherol) | ICH | MR Egger^b^ | 4 | -2.676 | 3.998 | 0.572 | -10.512 | 5.160 | 0.069 | 0.000 | 174.251 |
| Vitamin E (α-tocopherol) | ICH | Weighted median^c^ | 4 | 0.366 | 1.796 | 0.839 | -3.154 | 3.885 | 1.441 | 0.043 | 48.662 |
| Vitamin E (γ-tocopherol) | ICH | Inverse variance weighted | 5 | 1.206 | 1.052 | 0.252 | -0.855 | 3.268 | 3.340 | 0.425 | 26.249 |
| Vitamin E (γ-tocopherol) | ICH | MR Egger | 5 | 2.863 | 6.202 | 0.676 | -9.292 | 15.019 | 17.522 | 0.000 | 3330147.985 |
| Vitamin E (γ-tocopherol) | ICH | Weighted median | 5 | 1.070 | 1.270 | 0.400 | -1.419 | 3.558 | 2.914 | 0.242 | 35.088 |
| Zinc | ICH | Inverse variance weighted | 6 | -0.036 | 0.127 | 0.776 | -0.286 | 0.213 | 0.964 | 0.751 | 1.238 |
| Zinc | ICH | MR Egger | 6 | 0.114 | 0.462 | 0.817 | -0.791 | 1.019 | 1.121 | 0.454 | 2.771 |
| Zinc | ICH | Weighted median | 6 | 0.115 | 0.165 | 0.485 | -0.208 | 0.439 | 1.122 | 0.812 | 1.550 |
| Selenium | ICH | Inverse variance weighted | 5 | -0.043 | 0.150 | 0.773 | -0.337 | 0.250 | 0.958 | 0.714 | 1.284 |
| Selenium | ICH | MR Egger | 5 | 0.410 | 0.445 | 0.425 | -0.462 | 1.282 | 1.507 | 0.630 | 3.603 |
| Selenium | ICH | Weighted median | 5 | 0.131 | 0.185 | 0.477 | -0.231 | 0.494 | 1.141 | 0.794 | 1.639 |
| Carotene | ICH | Inverse variance weighted | 3 | -1.037 | 2.108 | 0.623 | -5.168 | 3.095 | 0.355 | 0.006 | 22.083 |
| Carotene | ICH | MR Egger | 3 | -2.907 | 4.568 | 0.639 | -11.860 | 6.045 | 0.055 | 0.000 | 422.208 |
| Carotene | ICH | Weighted median | 3 | 0.194 | 1.542 | 0.900 | -2.829 | 3.217 | 1.214 | 0.059 | 24.955 |
| Vitamin A (retinol) | SAH | Inverse variance weighted | 4 | -1.054 | 0.472 | 0.025 | -1.979 | -0.129 | 0.348 | 0.138 | 0.879 |
| Vitamin A (retinol) | SAH | MR Egger | 4 | -4.888 | 2.593 | 0.200 | -9.971 | 0.195 | 0.008 | 0.000 | 1.215 |
| Vitamin A (retinol) | SAH | Weighted median | 4 | -0.739 | 0.585 | 0.207 | -1.885 | 0.408 | 0.478 | 0.152 | 1.503 |
| Vitamin C (ascorbate) | SAH | Inverse variance weighted | 7 | -0.171 | 0.369 | 0.643 | -0.894 | 0.552 | 0.843 | 0.409 | 1.736 |
| Vitamin C (ascorbate) | SAH | MR Egger | 7 | 0.574 | 1.316 | 0.681 | -2.006 | 3.153 | 1.775 | 0.135 | 23.416 |
| Vitamin C (ascorbate) | SAH | Weighted median | 7 | -0.168 | 0.458 | 0.713 | -1.066 | 0.729 | 0.845 | 0.344 | 2.074 |
| Vitamin E (α-tocopherol) | SAH | Inverse variance weighted | 3 | -0.550 | 0.822 | 0.503 | -2.161 | 1.060 | 0.577 | 0.115 | 2.887 |
| Vitamin E (α-tocopherol) | SAH | MR Egger | 3 | -0.705 | 3.548 | 0.875 | -7.659 | 6.250 | 0.494 | 0.000 | 517.826 |
| Vitamin E (α-tocopherol) | SAH | Weighted median | 3 | -0.580 | 0.915 | 0.526 | -2.372 | 1.213 | 0.560 | 0.093 | 3.363 |
| Vitamin E (γ-tocopherol) | SAH | Inverse variance weighted | 4 | 0.633 | 0.579 | 0.274 | -0.502 | 1.769 | 1.884 | 0.605 | 5.863 |
| Vitamin E (γ-tocopherol) | SAH | MR Egger | 4 | 0.751 | 3.730 | 0.859 | -6.559 | 8.061 | 2.119 | 0.001 | 3169.075 |
| Vitamin E (γ-tocopherol) | SAH | Weighted median | 4 | 0.538 | 0.678 | 0.428 | -0.791 | 1.866 | 1.712 | 0.454 | 6.464 |
| Zinc | SAH | Inverse variance weighted | 6 | -0.004 | 0.062 | 0.945 | -0.126 | 0.118 | 0.996 | 0.881 | 1.125 |
| Zinc | SAH | MR Egger | 6 | -0.036 | 0.224 | 0.882 | -0.475 | 0.404 | 0.965 | 0.622 | 1.498 |
| Zinc | SAH | Weighted median | 6 | 0.030 | 0.077 | 0.701 | -0.121 | 0.180 | 1.030 | 0.886 | 1.198 |
| Selenium | SAH | Inverse variance weighted | 5 | -0.191 | 0.071 | 0.007 | -0.331 | -0.052 | 0.826 | 0.718 | 0.950 |
| Selenium | SAH | MR Egger | 5 | -0.007 | 0.215 | 0.977 | -0.428 | 0.415 | 0.993 | 0.652 | 1.514 |
| Selenium | SAH | Weighted median | 5 | -0.158 | 0.085 | 0.065 | -0.325 | 0.010 | 0.854 | 0.723 | 1.010 |
| Carotene | SAH | Inverse variance weighted | 5 | -0.763 | 0.829 | 0.358 | -2.388 | 0.863 | 0.466 | 0.092 | 2.371 |
| Carotene | SAH | MR Egger | 5 | -1.075 | 1.938 | 0.618 | -4.874 | 2.723 | 0.341 | 0.008 | 15.231 |
| Carotene | SAH | Weighted median | 5 | -0.395 | 0.663 | 0.551 | -1.695 | 0.904 | 0.673 | 0.184 | 2.469 |

^a^Inverse variance weighted (IVW): This method assumes that all IVs are valid instruments.

^b^MR Egger: This method assumes that all IVs are invalid instruments.

^c^Weighted median (WM): This method assumes that more than half of the IVs are valid instruments.

**Table S12** Sensitivity analysis of antioxidants on stroke.

| Exposeure | Outcome | Q | Q-df | *P*-Het | Egger-intercept | Egger-SE | *P*-Egger |
| --- | --- | --- | --- | --- | --- | --- | --- |
| Retinol | IS | 11.543 | 6 | 0.073 | 0.002 | 0.016 | 0.890 |
| Ascorbate | IS | 7.280 | 8 | 0.507 | -0.007 | 0.010 | 0.510 |
| α-tocopherol | IS | 2.236 | 3 | 0.525 | 0.015 | 0.016 | 0.464 |
| γ-tocopherol | IS | 7.123 | 5 | 0.212 | -0.022 | 0.017 | 0.271 |
| Zinc | IS | 4.882 | 6 | 0.559 | 0.017 | 0.013 | 0.249 |
| Selenium | IS | 1.279 | 5 | 0.937 | -0.002 | 0.011 | 0.839 |
| Carotene | IS | 8.153 | 10 | 0.614 | 0.003 | 0.008 | 0.720 |
| Retinol | SAH | 2.376 | 3 | 0.498 | 0.117 | 0.078 | 0.272 |
| Ascorbate | SAH | 3.547 | 6 | 0.738 | -0.026 | 0.044 | 0.581 |
| α-tocopherol | SAH | 0.026 | 2 | 0.987 | 0.004 | 0.095 | 0.972 |
| γ-tocopherol | SAH | 1.987 | 3 | 0.575 | -0.003 | 0.094 | 0.977 |
| Zinc | SAH | 2.006 | 5 | 0.848 | 0.007 | 0.047 | 0.892 |
| Selenium | SAH | 2.950 | 4 | 0.566 | -0.038 | 0.042 | 0.429 |
| Carotene | SAH | 14.366 | 4 | 0.006 | 0.014 | 0.075 | 0.865 |
| Retinol | ICH | 3.014 | 4 | 0.556 | -0.058 | 0.107 | 0.626 |
| Ascorbate | ICH | 5.529 | 5 | 0.355 | -0.122 | 0.076 | 0.183 |
| α-tocopherol | ICH | 1.897 | 3 | 0.594 | 0.125 | 0.119 | 0.404 |
| γ-tocopherol | ICH | 0.567 | 4 | 0.967 | -0.044 | 0.162 | 0.804 |
| Zinc | ICH | 4.177 | 4 | 0.383 | -0.033 | 0.097 | 0.751 |
| Selenium | ICH | 2.261 | 4 | 0.688 | -0.091 | 0.084 | 0.359 |
| Carotene | ICH | 7.343 | 2 | 0.025 | 0.106 | 0.211 | 0.703 |

‘*P*-Het’ represents the *P*-value belonging to the Q-statistic. ‘*P*-Egger’ represents the *P*-value belonging to the Egger-intercept.


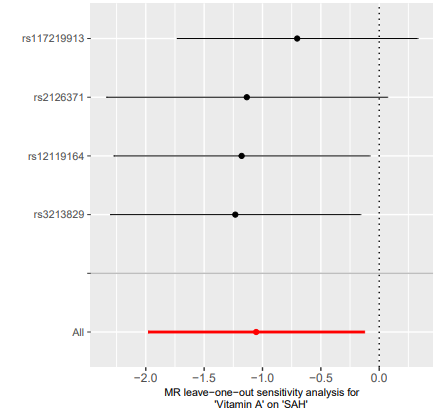


**Figure S3** Results of leave-one-out sensitivity analysis for Vitamin A on SAH.


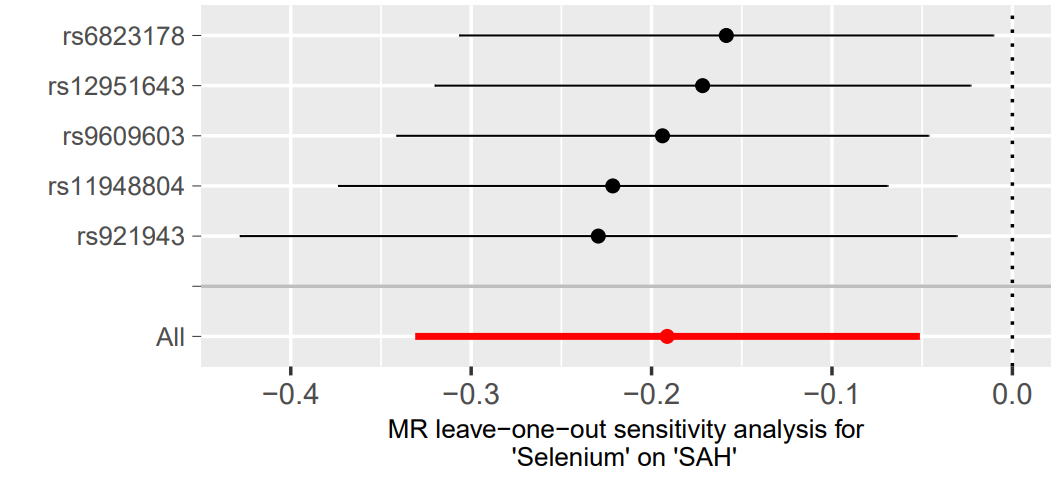


**Figure S4** Results of leave-one-out sensitivity analysis for Selenium on SAH.

## Reference

[1] SHIN S Y, FAUMAN E B, PETERSEN A K, et al. An atlas of genetic influences on human blood metabolites [J]. Nat Genet, 2014, 46(6): 543-50.

[2] EVANS D M, ZHU G, DY V, et al. Genome-wide association study identifies loci affecting blood copper, selenium and zinc [J]. Hum Mol Genet, 2013, 22(19): 3998-4006.

[3] Woo D, Falcone GJ, Devan WJ, et al. Meta-analysis of genome-wide association studies identifies 1q22 as a susceptibility locus for intracerebral hemorrhage. Am J Hum Genet 2014; 94: 511-521. 2014/03/25. DOI: 10.1016/j.ajhg.2014.02.012.

[4] BAKKER M K, VAN DER SPEK R A A, VAN RHEENEN W, et al. Genome-wide association study of intracranial aneurysms identifies 17 risk loci and genetic overlap with clinical risk factors [J]. Nat Genet, 2020, 52(12): 1303-13.

[5] Mishra A, Malik R, Hachiya T, et al. Stroke genetics informs drug discovery and risk prediction across ancestries. Nature 2022; 611: 115-123. 2022/10/01. DOI: 10.1038/s41586-022-05165-3..
